# Deciphering the role of DC subsets in MCMV infection to better understand immune protection against viral infections

**DOI:** 10.3389/fmicb.2014.00378

**Published:** 2014-07-29

**Authors:** Yannick O. Alexandre, Clément D. Cocita, Sonia Ghilas, Marc Dalod

**Affiliations:** ^1^Centre d'Immunologie de Marseille-Luminy, Aix-Marseille University, UM2Marseille, France; ^2^Institut National de la Santé et de la Recherche Médicale, U1104Marseille, France; ^3^Centre National de la Recherche Scientifique, UMR7280Marseille, France

**Keywords:** murine cytomegalovirus, plasmacytoid dendritic cells, XCR1+ dendritic cells, type I interferons, cross-presentation, NK cells, immune evasion, vaccination

## Abstract

Infection of mice with murine cytomegalovirus (MCMV) recapitulates many physiopathological characteristics of human CMV infection and enables studying the interactions between a virus and its natural host. Dendritic cells (DC) are mononuclear phagocytes linking innate and adaptive immunity which are both necessary for MCMV control. DC are critical for the induction of cellular immunity because they are uniquely efficient for the activation of naïve T cells during their first encounter with a pathogen. DC are equipped with a variety of innate immune recognition receptors (I2R2) allowing them to detect pathogens or infections and to engulf molecules, microorganisms or cellular debris. The combinatorial engagement of I2R2 during infections controls DC maturation and shapes their response in terms of cytokine production, activation of natural killer (NK) cells and functional polarization of T cells. Several DC subsets exist which express different arrays of I2R2 and are specialized in distinct functions. The study of MCMV infection helped deciphering the physiological roles of DC subsets and their molecular regulation. It allowed the identification and first *in vivo* studies of mouse plasmacytoid DC which produce high level of interferons-α/β early after infection. Despite its ability to infect DC and dampen their functions, MCMV induces very robust, efficient and long-lasting CD8 T cell responses. Their priming may rely on the unique ability of uninfected XCR1^+^ DC to cross-present engulfed viral antigens and thus to counter MCMV interference with antigen presentation. A balance appears to have been reached during co-evolution, allowing controlled replication of the virus for horizontal spread without pathological consequences for the immunocompetent host. We will discuss the role of the interplay between the virus and DC in setting this balance, and how advancing this knowledge further could help develop better vaccines against other intracellular infectious agents.

## Introduction

Human cytomegalovirus (HCMV) is DNA β-herpes virus extremely prevalent word wild and establishing a persistent latent infection in immunocompetent hosts. HCMV infection is generally asymptomatic even during primary infection. However, HCMV is an important opportunistic agent causing severe morbidity or death in immunocompromised hosts such as AIDS patients, fetuses and newborns (Krmpotic et al., 2003). To date, no vaccine exists against HCMV and designing one is a very active research field.

Mouse cytomegalovirus (MCMV) is a natural rodent pathogen, establishing life-long persistent latent infections in mice. *In natura*, MCMV is transmitted between mice mostly by biting or upon contact with feces of infected mice. These routes of infection are hard to mimic in laboratory. The corresponding physiological doses of virus inoculum are unknown. Inoculation of mice with relatively high doses of virus inoculum via the intra-peritoneal (i.p.) or intravenous (i.v.) routes are the main experimental models used. These experimental settings are highly reproducible and allow using many mutant mouse strains or viruses with reasonably low amounts of animals per group. This enabled to precisely decipher the cellular and molecular mechanisms regulating immune responses to MCMV. A parallel can be drawn for key pathological features and protection mechanisms between experimental MCMV infection in mice and *in natura* HCMV infection in humans. Hence, most of the observations gathered with experimental MCMV infection in mice should apply to some extent to *in natura* immune responses against MCMV infection in mice and most importantly against HCMV infection in humans.

During the acute phase of the infection, MCMV can infect hematopoietic cells including macrophages and dendritic cells (DC) but also many non-hematopoietic cells such as hepatocytes, endothelial cells or epithelial cells (Krmpotic et al., 2003). Both innate and adaptive immunity are required for resistance to MCMV infection. Amongst innate immune lymphocytes, Natural Killer (NK) cells are the most critical for defense against MCMV infection in several mouse strains. Indeed, NK cells can control MCMV replication by directly recognizing and killing infected cells, depending on the combined haplotypes of class I major histocompatibility (MHC-I) genes and NK cell receptor genes (Miletic et al., 2013). NK T cells (Van Dommelen et al., 2003; Tyznik et al., 2014) and γδ T lymphocytes (Ninomiya et al., 2000) can also contribute to innate immune defenses against MCMV infection. Adaptive humoral immunity mediates protection since antiviral antibodies efficiently limit viral replication during reactivation from latency (Jonjic et al., 1994) and antibody infusion can protect both adult and newborn mice from the pathology induced by MCMV infection (Cekinovic et al., 2008). CD8 T cell responses are critical for protection not only against acute infection in mouse strains with inefficient NK cell responses (Lathbury et al., 1996; Krmpotic et al., 1999) but also for long-term control of viral replication in all mouse strains by preventing the selection of innate immunity escape mutants (French et al., 2004, 2005) and by contributing to prevent viral reactivation from latency (Polic et al., 1998; Simon et al., 2006). CD4 T cell responses also play a key role in the pathophysiology of MCMV infection. During acute infection, antiviral CD4 T cells accumulate to high levels in the spleen and lungs where they produce both Th1 and Th17 cytokines (Arens et al., 2008; Walton et al., 2008). Through their IFN-γ secretion, CD4 T cells directly contribute to the control of viral replication in various organs (Walton et al., 2011a; Jeitziner et al., 2013) with a non-redundant role in the salivary glands (SG) where the functions of the other subsets of lymphocytes are compromised (Jonjic et al., 1989; Lathbury et al., 1996; Walton et al., 2011a; Thom et al., 2014). However, CD4 T cells can also contribute to immunosuppressive effects including IL-10 production which limits the induction of protective Th1 responses and promotes prolonged infection of the SG (Humphreys et al., 2007a; Mandaric et al., 2012). During the acute phase of the infection, CD4 T cell responses are critical to promote the induction of humoral immunity but dispensable for the induction of CD8 T cell responses (Jonjic et al., 1989). During the latent phase of the infection, CD4 T cell responses promote the expansion of antiviral CD8 T cells (Humphreys et al., 2007b; Snyder et al., 2009; Walton et al., 2011b). The activation of both NK and CD8 T cells heavily relies on their cross-talk with DC and many studies have investigated the underlying cellular and molecular mechanisms, including the role of specific DC subsets and/or specific DC functions. In contrast, much less is known on the interactions between DC and NK T cells, δγ T cells, CD4 T cells or B lymphocytes during MCMV infection. Hence, since our review is focused on the role of DC subsets during MCMV infection, we will discuss their impact on NK and CD8 T cell responses but not elaborate much on the functions of other lymphocyte populations. Specifically, we will develop the hypothesis that the interplay between DC and MCMV is critical in setting a delicate but mutually beneficial balance between the virus and its host. The health of the host is preserved despite the infection by two complementary mechanisms. First, viral replication and cytopathic effects are efficiently controlled in most organs by DC-dependent NK and CD8 T cell responses. Second, negative regulation of immune responses induced both by host feedback mechanisms and by virus manipulation of host cells prevents the development of severe immunopathology. The life cycle of the virus can be completed through establishment of latency and horizontal transfer to other hosts. We will discuss how viral immunoevasion genes and host professional cross-presenting XCR1^+^ DC play a key role in setting this balance, in particular by promoting the induction of protective inflationary effector memory CD8 T cell responses. This characteristic of adaptive immune responses to CMV infections is being used to help develop better vaccines against other intracellular infectious agents, notably Human Immunodeficiency Virus (HIV).

## A current simplified view on DC subset nomenclature and functional specialization

DC are rare heterogeneous mononuclear phagocyte cells of the immune system which are characterized by their unique ability to activate naïve T lymphocytes. DC can be classified in five main subsets mainly based on differences in their ontogeny and functions (Table 1) (Dalod et al., 2014). Plasmacytoid DC (pDC) and the two subsets of conventional DC (cDC): CD8α^+^-type DC and CD11b^+^ DC, share the same developmental pathway. They strongly depend on the growth factor FLT3-L for their differentiation and homeostasis. They derive from a proximal common hematopoietic progenitor, the common DC progenitor (CDP), which is devoid of any other differentiation potential. In contrast, Langerhans cells (LC), which are exclusively found in the epidermis, and monocyte derived DC (MoDC), which develop only during inflammation, belong to the monocytic lineage. Mouse pDC can be unequivocally identified as CD11b^−^CD11c^int^ and Bst2^hi^ or SiglecH^+^. They are specialized in rapid and high level production of the antiviral cytokines type I interferon (IFN-I), including during MCMV infection (Table 1). CD8α^+^-type DC and CD11b^+^ DC exist both as lymphoid tissue-resident DC (LT-DC) and as sentinel immune cells surveying all non-lymphoid tissues and migrating to their draining lymph nodes upon activation (MigDC). CD11b^+^ cDC are identified as Lineage ^−^CD11c^+/high^Ly6C^−^CD64^−^MerTK^−^ cells, to distinguish them from macrophages which express MerTK and from MoDC which derive from Ly6C^hi^ (classical) monocytes and are Ly6G^−^CD11b^+^CCR2^+^CD64^low^MerTK^−^ (Tamoutounour et al., 2013). CD8α^+^-type cDC can be identified as Lineage^−^SiglecH^−^CD11b^−^CD11c^+/high^ and either CD8α^+^, CD207^+^, CD24^+^ or CD103^+^ depending on the tissues and mouse strains examined. CD11b^+^ cDC are most efficient for CD4 T cell priming, in particular their polarization toward Th2 or Th17, and for the promotion of humoral immunity. They are considered to play a critical role in immune defenses against extracellular pathogens. CD8α^+^ cDC are most efficient for CD8 T cell priming, in particular through uptake and processing of exogenous antigens for their presentation in association with MHC-I molecules, a process called cross-presentation. CD8α^+^-type cDC are critical for immune defenses against cancer and against a variety of intracellular pathogens including MCMV (Table 1). Gene expression profiling and functional analyses have established homologies between mouse and human DC subsets, including between mouse CD8α^+^-type cDC and human CD141(BDCA3)^+^ cDC which both specifically express the chemokine receptor XCR1(Dorner et al., 2009; Bachem et al., 2010; Crozat et al., 2010, 2011). Hence, to promote a simple and universal DC subset nomenclature, in this review CD8α^+^-type cDC will be coined XCR1^+^ cDC. MoDC constitute only one of the differentiation fates of classical monocytes upon activation. Classical monocytes can also differentiate into inflammatory macrophages and myeloid-derived suppressor cells. It is not easy, and rarely achieved, to rigorously discriminate between activated classical monocytes, inflammatory macrophages, myeloid-derived suppressor cells and MoDC. The reader should keep this issue in mind when we will discuss the role of these cells in MCMV infection. Not much is known about the role of LC *in vivo* during MCMV infection. Hence, most of this review will focus on the role of pDC, cDC, and to some extent monocytes subsets and MoDC/activated classical monocytes/myeloid-derived suppressor cells.

**Table 1:** **Nomenclature, phenotype and *in vivo* functions of monocyte and DC subsets during MCMV infection**.

**Name**	**Markers**	**Functions during MCMV infection**			**References**
		Infection^*^	**Innate immunity**	**Adaptive Immunity**	
XCR1^+^ cDC^**^	Lin^−^ CD11b^−^ CD11c^high^ XCR1^+^ CD24^+^ CD8α^+/−^ CD103^+/−^	Yes, <5%	IL-12, IL-15 … NK cell activation	Priming of antiviral CD8 T cells in acute infection	Andrews et al., 2003; Dalod et al., 2003; Torti et al., 2011b; Busche et al., 2013
CD11b^+^ cDC	Lin^−^ CD64^−^ MerTK^−^CD11c^high^ CD11b^+^	Yes, <<1%	IL-12, IL-15 … NK cell activation	Control of CD4 T cell priming?	Dalod et al., 2003; Andoniou et al., 2005
pDC	Lin^−^ CD11b^−^ CD11c^int^ Bst2^+^ SiglecH^+^	No	IFN-I, IL-12, TNF, CCL3 … NK cell activation	Dispensable?	Asselin-Paturel et al., 2001; Dalod et al., 2002, 2003; Krug et al., 2004; Scheu et al., 2008; Zucchini et al., 2008a; Swiecki et al., 2010
Classical monocytes/MDSC/MoDC^***^	Lin^−^ MerTK^−^ CD11b^+^ Ly6C^high^ CCR2^+^ CD64^+/low^	No	iNOS, TNF, IL-15? … recruitment in the liver	Dampening of CD8 T cell responses to prevent immunopathology?	Daley-Bauer et al., 2012; Livingston-Rosanoff et al., 2012
Non-classical monocytes	Lin^−^ MerTK^−^ Ly6C^−/low^ CD11b^+^ CX3CR1^high^	Yes, dissemination of the virus, promotion of latent infection	?	?	Daley-Bauer et al., 2014
LC	Lin^−^ CD11c^+^ CD24^+^ CD11b^+^ CD207^high^	No	?	?	

* *In vivo viral infection of DC or monocyte subsets in mice challenged i.p. with MCMV*.

** *The name XCR1^+^ cDC is not yet an official nomenclature but has been coined here to define in a simple and general way all the CD8α^+^-type cDC of the mouse*.

*** *Studies do not always rigorously discriminate between activated classical monocytes, myeloid-derived suppressor cells (MDSC) and MoDC, which are therefore discussed together here*.

*^?^Contribution unknown*.

## Role of DC and monocytes in MCMV replication and dissemination

### What are the first cells infected by MCMV *in vivo*?

MCMV has a broad tropism. It can infect a variety of cell types *in vivo*, including neutrophils and many cells of the mononuclear phagocyte system (Krmpotic et al., 2003). Several studies analyzed the kinetics of MCMV replication and dissemination early after i.p. inoculation. Replicative virus is found in most visceral organs 1 week after infection. However, it needs cellular vehicles to disseminate in distal organs, in particular the SG, and to remain latent. To examine MCMV tropism *in vivo* at different times after i.p. infection, several studies used the same recombinant MCMV expressing the enhanced green fluorescent protein (EGFP) under control of the native immediate-early 1/3 promoter (Henry et al., 2000). In these experimental settings, among splenic DC subsets, XCR1^+^ DC were preferentially infected at 36–48 h after MCMV infection albeit at a very low frequency (~2–5% EGFP^+^) (Dalod et al., 2003). EGFP^+^ cells could be detected in several other hematopoietic cell types, prominently neutrophils (~15–20% EGFP^+^), also NK cells (3–7%) but not much B and T cells (<2%) (Banks et al., 2005; Benedict et al., 2006). However, viral replication occurred preferentially in non-hematopoietic, stromal, cells which constituted most of the source of viral RNA in the spleen at 3 days after infection (Benedict et al., 2006). Indeed, in the spleen, MCMV first infects stromal cells (ER-TR7^+^CD29^+^), in the marginal zone, at 6–8 h after inoculation. By 17 h, MCMV has disseminated in the red pulp, and by 48 h to the white pulp including in DC (Hsu et al., 2009). Hence, endothelial or fibroblastic stromal cells are the first targets infected by MCMV *in vivo*. They constitute the bulk of MCMV replicating cells in the spleen at all time points examined during acute infection of immunocompetent mice. A small proportion of different hematopoietic cell types is also infected *in vivo* relatively early after virus inoculation, including DC.

### Are DC or monocytes playing a significant role in MCMV replication and dissemination?

Splenectomized mice harbored decreased viral replication in the liver and increased survival upon acute MCMV infection (Katzenstein et al., 1983). Hence, the spleen appears to be an important site of MCMV replication early after systemic inoculation of MCMV and to promote dissemination to other organs. This suggests that specific populations of immune cells are essential to initiate MCMV infection and to promote its dissemination to the spleen and then throughout the body early after systemic inoculation of the virus, as was shown of XCR1^+^ DC for systemic infection with *Listeria monocytogenes* (Neuenhahn et al., 2006; Edelson et al., 2011). However, much remains to be done to evaluate the contribution of different subsets of DC or monocytes to MCMV replication and dissemination.

#### Contribution of DC and monocyte subsets to viral replication and spread across tissues during acute infection

To advance understanding of the mechanisms promoting MCMV dissemination *in vivo*, the group of Koszinowski has recently engineered a mutated strain of MCMV allowing to measure what fraction of the virus has been replicating in a given cell type (Sacher et al., 2008b). This “cell tropism-trap” MCMV encodes for a conditional reporter cassette consisting in the sequence coding for EGFP under the control of the HCMV major immediate-early promoter but downstream of a floxed transcriptional stop sequence. In the absence of expression of the Cre recombinase in infected cells, the viral progeny will remain unstained. However, after infection of a cell expressing Cre, the viral genome will have deleted the floxed stop sequence. All the cells consecutively infected by this virus progeny will be green. Thus, this system uniquely allows measuring the specific contribution of virtually any cell type to viral dissemination *in vivo*, by directly analyzing the fraction of EGFP-expressing virions in different organs at different times after infection. In CD11c-Cre transgenic mice, a considerable amount of EGFP^+^ virus was found in most organs. Thus, an important fraction of the virus has replicated *in vivo* at some time point in CD11c^+^ cells, likely in cDC (Sacher et al., 2008a). This observation supports the hypothesis that cDC may bear an important contribution to viral replication and spread across tissues during acute infection. Whether this phenomenon is necessary or redundant for the initiation of the infection or for viral dissemination across organs is unknown. To answer this question, it will be necessary to measure the impact of DC depletion at different times after infection on the viral load in different tissues. This could be achieved by using mice allowing conditional elimination of ZBTB46^+^ cells upon *in vivo* injection of diphtheria toxin (Meredith et al., 2012). It would also be interesting to define more precisely which subsets of CD11c^+^ cells contribute the most to MCMV replication *in vivo*. Among DC subsets, XCR1^+^ DC are preferentially infected by MCMV at 36–48 h after challenge (Dalod et al., 2003). However, XCR1^+^ DC isolated from the spleen at 18 h after challenge do not produce infectious virus contrary to CD11b^+^ DC (Busche et al., 2013). Viral titers are unaffected in the spleen and liver of Batf3^−/−^ mice, despite their lack of XCR1^+^ DC under steady state conditions (Torti et al., 2011b). This does not completely rule out a role of XCR1^+^ DC in viral dissemination, because inflammatory conditions promote redundancy in transcription factor expression and functions in the DC lineage, allowing Batf3-independent development of XCR1^+^ cDC (Tussiwand et al., 2012; Seillet et al., 2013) which has not been examined in MCMV infection. Moreover, in the rat model of CMV infection, the virus encodes for an XCR1 ligand which exclusively binds and attracts XCR1^+^ DC (Geyer et al., 2014). Hence, it would be interesting to rigorously address what is the contribution of XCR1^+^ DC to MCMV replication *in vivo*. This could be achieved by infecting *Xcr1*-Cre mice with the “cell tropism-trap” MCMV, or by evaluating the impact of XCR1^+^ DC depletion on MCMV spread to different tissues. Likewise to all mutant viruses engineered from the parental BAC pSM3fr MCMV, the “cell tropism-trap” MCMV bears a mutation in the MCK-2 ORF leading to expression of a truncated, nonfunctional, viral MCK-2 chemokine (Mitrovic et al., 2012a). MCK-2 attracts myeloid cells and participates to MCMV dissemination *in vivo* in particular to the SG (Fleming et al., 1999; Saederup et al., 2001). Thus, the *in vivo* dissemination of the “cell tropism-trap” MCMV must be altered. Therefore, it would be important to engineer an MCK-2-repaired “cell tropism-trap” MCMV for future studies.

#### Role of DC and monocyte subsets for viral dissemination to salivary glands and for establishment of latency

After footpad inoculation of MCMV, the viral chemokine MCK-2 recruits non-classical (CX3CR1^hi^ patrolling) monocytes to infection site where they become infected. These cells then serve as immune vehicles to disseminate the virus to distal organs, especially to the SG. Non-classical monocytes are also essential for the establishment of latent infection (Daley-Bauer et al., 2014). However, this does exclude a contribution of DC subsets or other cell types to this process.

## DC ability to induce protective antiviral CD8 T cell responses is differentially impacted by *in vitro* vs. *in vivo* infections

**Figure 1:**
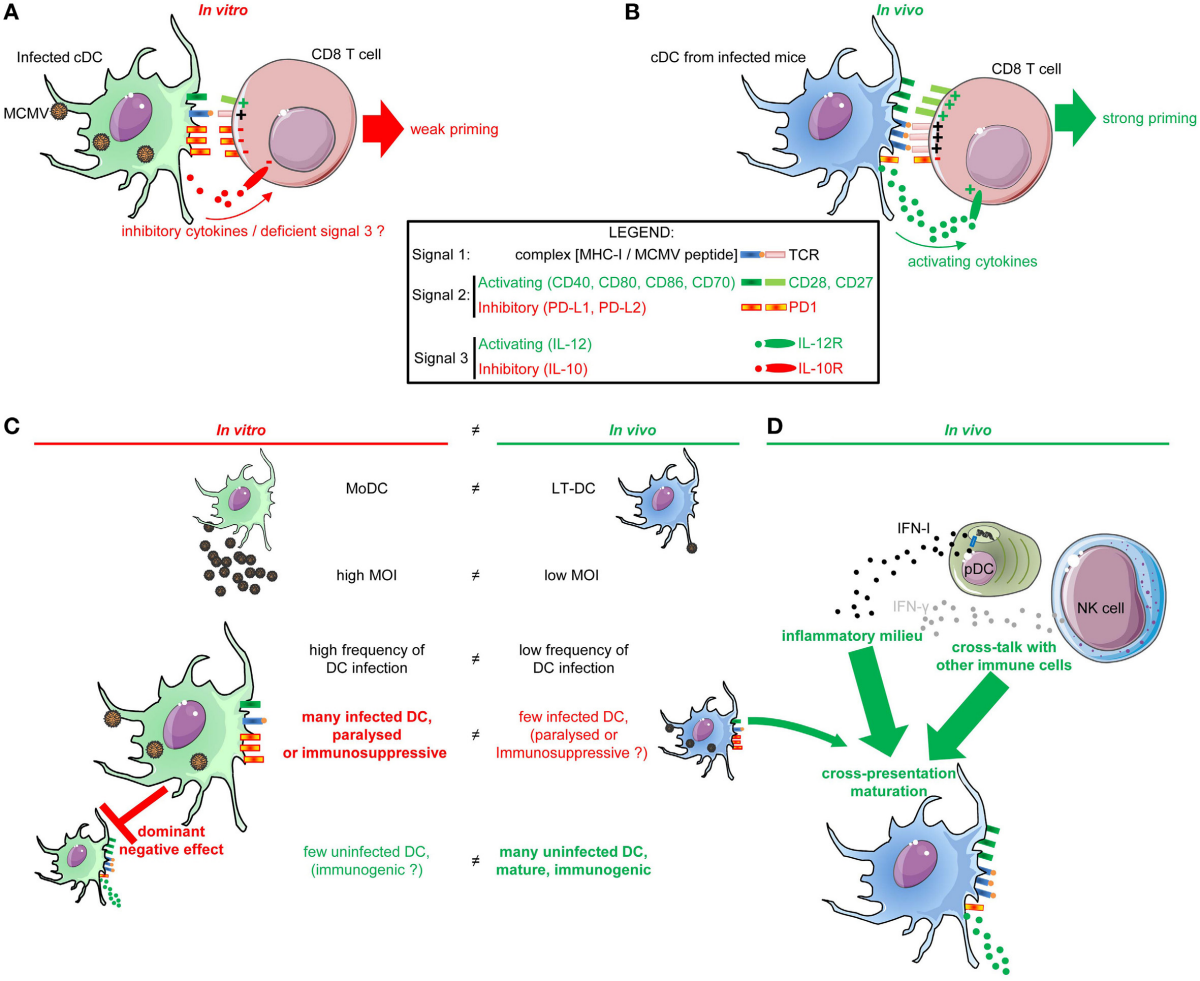
**DC functions are differentially impacted by their infection *in vitro* vs. the infection of mice *in vivo*. (A)** Impact on DC functions of their infection by MCMV *in vitro*. *In vitro*, MCMV-infected DC are “paralyzed” by the virus which prevents them to deliver adequate signals 1 and 2 for antiviral CD8 T cell activation. Specifically, infected DC down-regulate their expression of MHC-I and activating co-stimulation (CD40, CD80, CD86) molecules as a consequence of their expression of viral immune evasion genes. They induce inhibitory co-stimulation molecules (PD-L1 and PD-L2). Hence, infected DC only weakly prime antiviral CD8 T cells. **(B)** cDC functions *in vivo* in MCMV-infected mice. cDC are very strongly activated by MCMV infection *in vivo*, in a way enabling them to induce potent T cell activation *in vitro*, and consistent with the fact that MCMV infection induces very strong and protective antiviral cellular immune responses *in vivo*. Specifically, XCR1^+^ DC and CD11b^+^ cDC strongly up-regulate MHC and activating co-stimulation molecules *in vivo*, and can also produce T cell-activating cytokines such as IL-12 and IL-15. **(C,D)** Proposed explanations to the discrepancy of the impact of MCMV infection on DC *in vitro* vs. *in vivo*. **(C)** Impact of the nature of DC subsets and of their frequency of infection. The DC used *in vitro* are derived from monocytes (MoDC) and strongly differ from the DC involved *in vivo* in the induction of anti-viral immune responses in lymphoid tissues (LT-DC). High MOI are used for *in vitro* infection of DC, leading to a very high proportion of infected cells subjected to the effects of viral immune evasion genes. In contrast, only a very small fraction of XCR1^+^ cDC and CD11b^+^ cDC is infected *in vivo*. **(D)** Protective functions of cDC in MCMV infected mice are promoted by the inflammatory milieu, in particular by IFN-I and IFN-γ, and by cross-talk with other immune cells.

MCMV encodes for many immune evasion genes, including some genes compromising MHC-I antigen presentation by infected cells. However, contradictory reports have been published regarding the ability of the virus to paralyze DC functions. We will discuss how these reports could be reconciled by taking into account differences in the interactions between MCMV and DC *in vitro* vs. *in vivo* (Figure 1).

### *In vitro* infection of DC by MCMV compromises their ability to induce antiviral adaptive immunity

DC lines or MoDC derived *in vitro* in GM-CSF bone marrow cultures have been extensively used to investigate the interactions between MCMV and DC (Andrews et al., 2001; Mathys et al., 2003; Loewendorf et al., 2004; Mintern et al., 2006; Benedict et al., 2008). These studies showed that DC are permissive to MCMV *in vitro*, and that infected DC are relatively poor activator of T cells (Andrews et al., 2001; Mathys et al., 2003; Benedict et al., 2008) (Figure 1A). A variety of viral immune evasion genes affect the delivery by infected DC to T cells of all the three types of signals necessary for their priming. Signal 1 corresponds to the triggering of the T cell receptor by viral peptide-MHC-I complexes. Signal 2 corresponds to the triggering of activating co-stimulation receptor such as CD28 or CD27 by the CD80/86 and CD70 co-stimulation molecules induced on mature DC. Signal 3 corresponds to cytokines which can contribute to promote the proliferation of T cells and instruct their differentiation toward specific functions.

#### Inhibition of signal 1 delivery to T cells: inhibition of antigen processing and presentation in DC infected by MCMV in vitro

DC infected *in vitro* by MCMV have lower expression of MHC-I and MHC-II molecules at their surface as compared to non-infected DC (Andrews et al., 2001; Benedict et al., 2008) (Figure 1A). Three MCMV genes have been identified to regulate MHC-I expression on infected cells and DC: m04, m06, and m152, collectively called MHC-I immune evasion genes. These three genes inhibit the transport of MHC-I molecules to the cell surface, but also block the transporter associated with antigen processing machinery. This leads to a drastic reduction of cell surface expression of viral peptide-MHC-I complexes on infected DC and therefore to a strong impairment of their ability to deliver signal 1 for the activation of antiviral CD8 T cells (Hengel et al., 1999).

#### Modulation of signal 2 delivery to T cells: inhibition of the expression of activating co-stimulation molecules and induction of the expression of inhibitory co-stimulation molecules on DC infected by MCMV in vitro

The viral m147.5 gene specifically blocks cell surface export of CD86 and m138 blocks CD80 (Loewendorf et al., 2004; Mintern et al., 2006). These MCMV genes do not affect globally the secretory pathway of infected cells, because cell surface export of other molecules is not affected. In DC infected *in vitro*, m138 co-localizes with CD80 in lysosomes, suggesting a direct interaction between these two molecules leading to a redirection of the intracellular trafficking of CD80 (Mintern et al., 2006). The viral m155 gene inhibits CD40 expression on *in vitro* infected DC, possibly by inhibit the translation of *Cd40* mRNA (Loewendorf et al., 2011). As a result, DC infected *in vitro* by MCMV express low amounts of the activating co-stimulation molecules CD40, CD80, and CD86 (Figure 1A). Infected DC also up-regulate the inhibitory co-stimulation molecules PD-L1 and PD-L2 (Andrews et al., 2001; Mathys et al., 2003; Benedict et al., 2008). Thus, *in vitro* infection of DC by MCMV biases the nature of the signal 2 that they deliver to CD8 T cells toward inhibition (Figure 1A).

#### Modulation of signal 3 delivery to T cells: how does in vitro infection of DC by MCMV impact their cytokine production?

Little is known about the role of viral immune evasion genes in modulating DC cytokine responses. A study on infected macrophages showed that the viral *ie1* gene blocks their capacity to produce TNF through a pathway independent of NFKB (Rodriguez-Martin et al., 2012). The viral m45 gene blocks the NFKB pathway in infected fibroblast, resulting in a loss of TNF production (Mack et al., 2008). The m45 gene also blocks TLR3 signaling in infected fibroblasts (Mack et al., 2008). It is possible that *in vitro* infection of DC by MCMV also affects their expression of cytokines, to prevent the delivery of an activating signal 3 to antiviral CD8 T cells or even to induce the release of an inhibitory signal 3 such as IL-10 (Figure 1A).

### The immense majority of DC from MCMV infected mice undergo a strong immunogenic maturation and are competent for the induction of antiviral adaptive immunity

In contrast to what is observed upon *in vitro* infection of DC lines or MoDC, mouse splenic DC subsets undergo a strong immunogenic maturation *in vivo* in the spleen between 36 and 48 h after infection (Figure 1B). This was shown through measurements of different parameters, including their expression of cytokines and of MHC and co-stimulation molecules (Dalod et al., 2002, 2003; Delale et al., 2005; Zucchini et al., 2008a; Baranek et al., 2012), by gene expression profiling studies (Baranek et al., 2012; Vu Manh et al., 2013), and by functional assays (Dalod et al., 2003). DC isolated *ex vivo* after MCMV infection activate more efficiently naïve CD8 T cells as compared to DC isolated from non-infected mice, likely due to their activation state (Dalod et al., 2003) (Figure 1B).

#### Competence of DC isolated from MCMV-infected mice for signal 1 delivery to T cells

CD11b^+^ DC isolated from infected C57BL/6 mice are productively infected and fail to activate naïve anti-MCMV CD8 T cells in *ex vivo* culture, suggesting that productive MCMV infection of CD11b^+^ DC *in vivo* compromises their ability to directly process and present endogenously synthesized viral antigens to CD8 T cells (Busche et al., 2013). However, most splenic DC strongly up-regulate MHC-I (Figure 1B) and MHC-II molecules at their surface early after MCMV infection (Dalod et al., 2003; Delale et al., 2005). In addition, XCR1^+^ DC isolated from infected mice efficiently present *in vivo* acquired MCMV antigens to CD8 T cells (Busche et al., 2013). Thus, contrary to *in vitro* infected DC lines or MoDC, a significant fraction of LT-DC isolated from MCMV-infected mice is competent for signal 1 delivery to T cells (Figure 1B).

#### Competence of DC isolated from MCMV-infected mice for activating signal 2 delivery to T cells

During MCMV infection, most splenic DC strongly up-regulate CD40, CD80, and CD86 at their surface (Figure 1B) (Dalod et al., 2003; Delale et al., 2005). In addition, DC subsets isolated from infected mice and pulsed *ex vivo* with optimal epitopic peptides better activate cognate CD8 T cells than DC isolated from uninfected animals (Dalod et al., 2003). Thus, contrary to *in vitro* infected DC lines or MoDC, LT-DC isolated from MCMV-infected mice are competent for signal 2 delivery to T cells. Even though splenic DC from infected mice do up-regulate the inhibitory co-stimulation molecule PD-L1 (Figure 1B) (Baranek et al., 2012; Vu Manh et al., 2013), this does not appear to strongly impact on their ability to activate antiviral CD8 T cells. Indeed, *in vivo* blockade of PD1 only marginally increases antiviral CD8 T cell responses which are already very robust in the absence of treatment (Benedict et al., 2008).

#### Competence of DC isolated from MCMV-infected mice for signal 3 delivery to induce protective T cell functional polarization

Many innate cytokines and chemokines are produced early after MCMV infection *in vivo*, around 36 h after challenge (Ruzek et al., 1997), including IFN-I, IL-15, and IL-12 which are critical for antiviral defense (Orange and Biron, 1996a,b; Presti et al., 1998; Nguyen et al., 2002; Strobl et al., 2005; Baranek et al., 2012). The receptor for IFN-I is expressed ubiquitously and it is likely that the main function of IFN-I is to induce cell-intrinsic antiviral effector molecules in uninfected cells to enable them to resist MCMV infection. The nature of IFN-I induced genes conferring cell-intrinsic immunity to MCMV is unknown. Of note, MCMV encodes for an immune evasion gene inhibiting IFN-I and IFN-γ responses in infected cells by targeting the downstream signaling molecule STAT2 (Zimmermann et al., 2005). However, IFN-I also mediates immunoregulatory functions which are critical for the control of MCMV infection. More than a decade ago, the study of MCMV infection allowed the identification and first *in vivo* studies of mouse pDC, by demonstrating that they are the major producers of IFN-I early after infection (Asselin-Paturel et al., 2001; Dalod et al., 2002). This has since been confirmed by several independent studies (Krug et al., 2004; Scheu et al., 2008; Swiecki et al., 2010). More generally, during MCMV infection, a fraction of splenic DC produce IFN-I, IL-12, and IL-15 (Dalod et al., 2002, 2003; Zucchini et al., 2008a; Vu Manh et al., 2013). Hence, DC isolated from MCMV-infected mice are competent for signal 3 delivery to induce protective T cell functional polarization (Figure 1B).

### How to explain the discrepancy of the impact of MCMV infection on DC *in vitro* vs. *in vivo*?

Several factors can contribute to the discrepancy of the impact of MCMV infection on DC *in vitro* vs. *in vivo* (Figures 1C,D).

#### The DC subsets generally used for *in vitro* studies are different from LT-DC

The DC subsets generally used for *in vitro* studies belong to the monocyte lineage and are not equivalent to the LT-DC subsets responsible for the induction of adaptive immunity in secondary lymphoid organs (Figure 1C) (Robbins et al., 2008). Hence, these *in vitro* models might not faithfully represent the *in vivo* interplay between MCMV and DC. Indeed, it was recently shown that, upon infection with MCMV *in vitro*, MoDC but not FLT3-L DC fail to produce IL-12 and to activate NK T cells, while both uninfected DC subsets are able to produce the cytokine upon stimulation with a synthetic TLR9 ligand (Tyznik et al., 2014). Spleen DC subsets exposed to MCMV *in vitro* produce IL-12 (Krug et al., 2004) likewise to their *in vitro* derived FLT3-L DC counterparts and contrary to MoDC. These observations suggest that some of the differences observed regarding the consequences of DC exposure to MCMV *in vitro* vs. *in vivo* might result from different cell-intrinsic properties of MoDC and LT-DC, including maybe a higher susceptibility of the former to some of the effects of the virus immunoevasion genes. Hence, it will be interesting to examine whether MCMV infection differentially affect *in vivo* DC from the monocytic lineage, namely LC and MoDC, as compared to LT-DC. However, as seen with DC lines or MoDC, LT-DC isolated from uninfected mice and exposed *in vitro* to MCMV do show a strong impairment in their induction of activating co-stimulation molecules, strongly up-regulate inhibitory co-stimulation molecules, and fail to efficiently activate antiviral CD8 T cells *in vitro* as well as upon *in vivo* transfer (Benedict et al., 2008; Busche et al., 2013). Hence, it is likely that the LT-DC infected by MCMV *in vivo* are paralyzed or even exert immunosuppressive functions (Figure 1C). Thus, additional factors must account for the ability of LT-DC to evade *in vivo* the immunosuppressive functions of MCMV.

#### Only a very small fraction of DC are infected by MCMV *in vivo* and hence potentially susceptible to the action of viral immune evasion genes

High MOI are used for *in vitro* infection of DC, leading to a very high proportion of infected cells paralyzed or polarized toward immunosuppressive functions due to cell-intrinsic effects of viral immune evasion genes (Figure 1C). This may also lead to a strong dominant negative effect of these infected DC over the action of the few uninfected immunogenic DC eventually present in the culture (Figure 1C). In contrast, *in vivo*, in tissues, the ratio of the numbers of infectious viral particles to the numbers of DC is low (low MOI) (Figure 1C). Moreover, the probability of encounter between the virus and DC *in vivo* is lower due to the complexity of the tissue environment. Indeed, only a very small fraction of XCR1^+^ cDC and CD11b^+^ cDC is infected *in vivo* (Figure 1C). pDC are not infected. The first study that had examined the impact of MCMV infection on DC *in vivo* claimed that a major fraction of the splenic DC is infected and that this leads to a global paralysis of DC functions (Andrews et al., 2001). However, all of the other studies that have since examined MCMV tropism for DC *in vivo* reported that only a very minor fraction—a few percent—of splenic DC are infected at any time point after challenge (Dalod et al., 2003; Banks et al., 2005; Benedict et al., 2006; Mitrovic et al., 2012a). Moreover, all these later studies concur that DC are strongly activated by MCMV infection *in vivo* in a way enabling them to induce potent T cell activation *in vitro*, and consistent with the fact that MCMV infection induces very strong and protective antiviral cellular immune responses *in vivo*. However, CD11b^+^ cDC isolated from the spleen of infected mice produce infectious viral particles and fail to activate antiviral CD8 T cells *ex vivo*, unless they are exogenously loaded with synthetic viral epitopic peptides (Busche et al., 2013). Moreover, most of the DC infected by MCMV *in vivo* do not produce detectable levels of activation cytokines, not only IFN-I but also IL-12 (Dalod et al., 2003). This suggests that the cDC that are infected by MCMV *in vivo* are “paralyzed” by the virus at least for antigen processing and presentation, and for T-cell activating cytokine production, i.e., for signal 1 and 3 delivery (Figure 1C). The expression of co-stimulation molecules (signal 2) on the DC infected by MCMV *in vivo* has not yet been reported. The consequences on the functions of XCR1^+^ DC of their infection *in vivo* are not clear, although it may not result in the production of infectious virus (Busche et al., 2013). It would be interesting to purify infected (EGFP^+^) and non-infected (EGFP^−^) splenic DC subsets from the same mice and examine their gene expression profiling in order to get an unbiased and global view of how the infection of DC *in vivo* modulates their biology. In any case, *in vivo*, most DC are not infected and thus not susceptible to the cell-intrinsic action of viral immune evasion genes. In addition, there is no dominant negative effect of the DC that are eventually paralyzed or polarized toward immunosuppressive functions due to their infection by MCMV. On the contrary, *in vivo*, most of the DC are uninfected, mature and immunogenic (Figure 1C).

#### Protective functions of DC in MCMV infected mice are promoted by the inflammatory milieu and by cross-talk between different immune cell types

Other major factors likely contributing to explain the discrepancy of the impact of MCMV infection on DC *in vitro* vs. *in vivo* are differences in the microenvironment in which the interaction between the virus and the DC is taking place. We will discuss in particular the role of the inflammatory milieu and of cross-talk with other immune cell types in promoting protective functions of DC in MCMV infected mice (Figure 1D).

**Figure 2:**
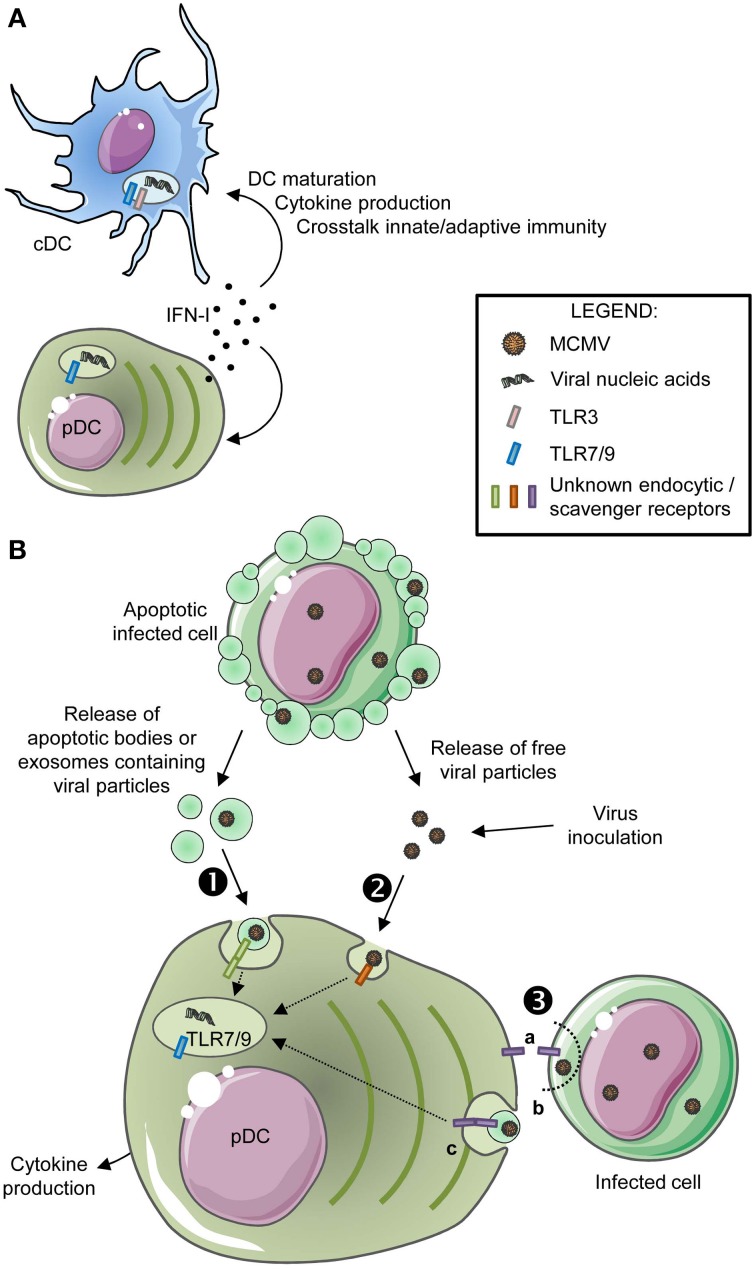
**Molecular mechanisms regulating DC subset activation during MCMV infection. (A)** Mechanism promoting DC subset maturation *in vivo* during MCMV infection. High amount of IFN-I are produced *in vivo* by pDC early after MCMV infection, which drives broad cell-intrinsic responses in all DC, including canonical DC maturation, promoting protective crosstalk with innate and adaptive immune cells. **(B)** Mechanisms promoting pDC sensing of MCMV infection. Early after completion of the first cycle of virus replication *in vivo*, pDC sense MCMV nucleotide sequences via endosomal TLRs, which leads to innate cytokine production, in particular IFN-I. How MCMV material ends up in the endosomes of pDC is still not understood. pDC might specifically recognize and engulf materials derived from infected cells and containing viral nucleic acids, either apoptotic bodies or exosomes (❶). pDC may also be able to detect and engulf MCMV particles released by infected cells or even from the viral inoculum (❷). Finally, pDC may directly sense and nibble infected cells (❸).

***Role of cell intrinsic responses of DC to IFN-I in promoting protection against MCMV infection***. Mice deficient for the alpha chain of the receptor for IFN-I (IFNAR) harbor a 7-fold increase in the fraction of infected DC, showing that IFN-I efficiently protects DC from MCMV infection *in vivo* (Dalod et al., 2003). Moreover, IFN-I also strongly promotes the maturation of DC subsets during MCMV infection *in vivo*, since splenic DC isolated from IFNAR^−/−^ mice show a drastic impairment in their induction of MHC-I and activating co-stimulation molecules (Dalod et al., 2003), which is due to cell-intrinsic IFN-I responses in DC (Baranek et al., 2012) (Figures 1D, 2A). More generally, cell-intrinsic effects of IFN-I in DC strongly contribute to drive their broad gene expression reprogramming during MCMV infection, and are essential to promote protection against disease and death, while it is not the case in NK cells (Baranek et al., 2012). It is not yet known precisely what protective functions of DC are promoted by IFN-I during MCMV infection, in which DC subset, and how. These questions should be possible to address in the near future by studying the impact of conditional genetic inactivation of IFNAR in specific DC subsets, as can be achieved by crossbreeding IFNAR-floxed mice with mice expressing the Cre recombinase in a specific DC subset.

***Role of NK cell responses in preserving DC numbers and functions during MCMV infection***. Mice that fail to control early replication of MCMV due to lack of efficient antiviral NK cell activity show a dramatic decrease in the numbers of XCR1^+^ DC in the spleen (Andoniou et al., 2005; Robbins et al., 2007; Mitrovic et al., 2012a). Hence, NK cell activity contributes to preserve DC homeostasis and likely promotes protective DC functions *in vivo* during MCMV infection. This is likely due in part to indirect effects linked to efficient early control of viral replication (Robbins et al., 2007) but it may also involve a direct cross-talk between NK cells and XCR1^+^ DC which remains to be examined (Figure 1D). In addition, NK cell delivery of IFN-γ to DC also likely contributes to promote their maturation (Figure 1D), not only for MHC-II expression but also potentially for IL-12 production.

In conclusion, *in vitro* studies have been useful to dissect the interplay between MCMV and DC (Figure 1A), but it should be paid attention that, *in vivo*, most DC are not infected, are mature, and are competent for *ex vivo* activation of CD8 T cells (Figures 1B–D). Hence, it is now quite clear that there is no induction of global DC functional paralysis by MCMV *in vivo*. The small fraction of the DC that are infected by MCMV does not exert strong dominant immunosuppressive functions *in vivo*. They may only slightly reduce the activation of CD8 T cells due to their expression of the inhibitory co-stimulation molecule PD-L1 (Benedict et al., 2008). MCMV infection very potently activates DC *in vivo*, in part due to the induction of high levels of IFN-I which promote DC maturation and protective functions against disease and death. It is likely that this remarkable activation of DC *in vivo* by MCMV bears a significant contribution to the balance that has been reached between this virus and its host during millions of years of co-evolution, to preserve health in immunocompetent hosts while still allowing establishment of viral latency and horizontal virus transfer.

## Molecular mechanisms regulating DC subset sensing of, and cytokine production in response to, MCMV

### Role of toll-like receptors in MCMV sensing by DC subsets

The peak of systemic production of IFN-I and IL-12 occurs between 30 and 48 h after MCMV infection. pDC are the main producers of these cytokines (Figure 1D) (Dalod et al., 2002; Zucchini et al., 2008a), although they are not infected (Dalod et al., 2003). IFN-I and IL-12 production, and more generally immune defenses against MCMV infection *in vivo*, are compromised in mice deficient for Toll-like receptor 9 (TLR9) or for the downstream signaling adaptor MyD88, as well as to a lesser extent in mice deficient for TLR3 or for the downstream signaling adaptor TRIF (Krug et al., 2004; Tabeta et al., 2004; Delale et al., 2005; Zucchini et al., 2008b). Indeed, pDC sensing of MCMV infection is completely dependent on MyD88, with a major contribution of TLR9 but a partial redundancy with TLR7 (Krug et al., 2004; Tabeta et al., 2004; Delale et al., 2005; Zucchini et al., 2008b). TLR9 recognizes unmethylated CpG DNA sequences that are absent in the genome of mammals. TLR7 recognizes single stranded RNA and TLR3 double stranded RNA. TLR3, 7, and 9 are located in endosomes and are thus not exposed to host nucleic acids. Hence, MCMV sensing by pDC must require active capture and endocytosis of viral particles or materials derived from infected cells (Figure 2B).

### Sources and mode of delivery of MCMV-derived nucleic acids to pDC

How pDC detect the virus before its entry in the endosomal compartment is poorly understood and could occur through distinct but not necessarily exclusive mechanisms (Figure 2B). It is likely that, *in vivo*, pDC recognize and engulf apoptotic bodies or exosomes released by MCMV-infected cells, (Figure 2B, ❶), as was demonstrated for other viruses (Dreux et al., 2012) or tumor cells (Bastos-Amador et al., 2012). pDC could also directly recognize and engulf viral particles (Figure 2B, ❷). It is also possible that direct transfer of viral material occurs from live infected cells to pDC, through cell-cell contacts (Figure 2B, ❸), including by nibbling of the infected cells by pDC as shown *in vitro* in co-cultures of human pDC and HSV-1-infected MoDC (Megjugorac et al., 2007). The I2R2s allowing pDC to recognize and/or engulf viral particles or material from infected cells are still unknown. Their identification is under intensive investigation. The endocytic C-type lectin receptor SiglecH, which was considered as a likely candidate, is dispensable for this function (Puttur et al., 2013).

### Modulation of DC subset responses during MCMV infection by other I2R2s

During MCMV infection, several I2R2s modulate DC cytokine production positively or negatively. SiglecH inhibits IFN-I production during MCMV infection *in vivo* and can inhibit pDC IFN-I production upon exposure to MCMV *in vitro* (Blasius et al., 2006). However, a large part of the inhibitory effect of SiglecH on IFN-I production *in vivo* during MCMV infection occurs in other cells than pDC (Swiecki et al., 2014). Moreover, although SiglecH^−/−^ mice show significant increases in their systemic levels of IFN-I during MCMV infection, this does not impact on the control of viral replication and on morbidity or death (Puttur et al., 2013). Contrary to SiglecH, the C-type lectin Ly49Q promotes higher production of IFN-I by pDC during exposure to MCMV *in vitro*, through recognition of MHC-I (Tai et al., 2008). However, no such effect is observed *in vivo* in Ly49Q^−/−^ mice, where a slight reduction in serum levels of IL-12 is observed in correlation to a moderate increase in viral loads in spleen. Hence, the biological significance of SiglecH and Ly49Q expression on pDC for the modulation of their *in vivo* responses to viral infections remains an enigma. Finally, during MCMV infection *in vivo*, IFN-I inhibits IL-12 production by CD11b^+^ and XCR1^+^ DC (Dalod et al., 2003; Krug et al., 2004; Swiecki et al., 2010). This might constitute a safeguard mechanism to prevent excessive production of IL-12 and its immunopathological consequences.

## Role of DC subsets in the regulation of NK cell responses to MCMV infection

NK cell responses were first demonstrated to be critical for resistance to MCMV infection more than 30 years ago (Bancroft et al., 1981; Shellam et al., 1981). A large set of genetically modified mouse and virus strains allowed a precise understanding of the interplay between NK cells and MCMV (Miletic et al., 2013). Here, we will focus on cross-talk between DC and NK cells. More specifically, we will discuss the contribution of DC subsets to the cytokine-mediated activation of all NK cells and to the induction of the proliferation of the subset of NK cells specifically able to recognize MCMV-infected cells.

### Role of DC subsets in “aspecific” NK cell acquisition of their antiviral effector molecular machinery early after *in vivo* infection

During MCMV infection, IL-12 promotes NK cell IFN-γ production, while IFN-I-induced IL-15 promotes NK cell proliferation, survival and cytotoxicity (Orange and Biron, 1996b; Nguyen et al., 2002; Baranek et al., 2012). Since DC are the major source of IL-12 and IFN-I, they should significantly contribute to the cytokine-dependent induction of antiviral effector molecules in NK cells, early after *in vivo* infection. However, *in vivo* depletion of pDC did not dampen NK cell activation but rather enhanced it, irrespective of the method used [injection of anti-Ly6G (Dalod et al., 2002) or anti-PDCA-1/Bst2/120G8 antibodies (Krug et al., 2004) or diphtheria toxin injection in mice transgenic for the expression of hDTR under the control of the promoter of the human *CLEC4C* gene (Swiecki et al., 2010)]. This was correlated to an exacerbated production of IL-12 by cDC (Dalod et al., 2002; Krug et al., 2004; Swiecki et al., 2010). Hence, pDC appear to be dispensable for efficient cytokine-dependent activation of NK cells *in vivo* during MCMV infection. This may be due to redundancy between the functions of pDC and those of cDC. NK cells only express the low avidity receptor for IL-15, IL-15Rβ /γ, while myeloid cells express the high affinity chain of this receptor, IL-15Rα. As a result, efficient activation of NK cells by low, physiological, doses of IL-15 requires trans-presentation of the cytokine on IL-15Rα by myeloid cells (Burkett et al., 2004; Lucas et al., 2007; Mortier et al., 2008). pDC express only very low levels of the genes encoding IL-15 and IL-15Rα in MCMV-infected mice, contrary to cDC (Baranek et al., 2009, 2012). However, in addition to cDC, other cell types could contribute to trans-present IL-15 to NK cells, including MoDC, monocytes, macrophages and/or stromal cells (Mortier et al., 2009; Cui et al., 2014). Hence, the precise delineation of the role of cDC for cytokine-dependent NK cell activation during MCMV infection will require investigating the impact on NK cell responses of the depletion of cDC alone or both cDC and pDC. It would also be informative to genetically inactivate cytokine production specifically in DC subsets.

### Role of DC subsets in the “cognate” NK cell proliferation later after *in vivo* infection

C57BL/6 mice are much more resistant to MCMV infection than BALB/c mice. This is due to a single locus, *Cmv1*, located in the NK gene complex which encompasses the genes encoding the Ly49 activation and inhibitory C-type lectin NK cell receptors (Scalzo et al., 1990). *Cmv1* codes for the Ly49H activation NK cell receptor (Brown et al., 2001; Daniels et al., 2001; Lee et al., 2001). Ly49H specifically binds an MCMV-encoded protein, m157, expressed at the surface of infected cells (Arase et al., 2002; Smith et al., 2002; Bubic et al., 2004). Engagement of Ly49H by m157 triggers cytotoxic granules exocytosis and is necessary for NK cell recognition and killing of MCMV-infected cells in C57BL/6 mice. In addition, engagement of Ly49H by m157 is sufficient to promote the production of IFN-γ and the proliferation of Ly49H-expressing NK cells *in vitro* in the absence of NK cell-activating cytokines (Arase et al., 2002; Smith et al., 2002; Bubic et al., 2004). *In vivo*, a specific expansion of Ly49H^+^ NK cells is observed at late time points after MCMV infection (Dokun et al., 2001). It leads to the induction of anti-MCMV NK cell memory (Sun et al., 2009a), which depends at least in part on the NK cell-activating cytokines IL-15 and/or IL-12 on the one hand (Andrews et al., 2003; French et al., 2006; Sun et al., 2009b, 2012; Firth et al., 2013) and on XCR1^+^ DC on the other hand (Andrews et al., 2003). Which cells deliver IL-12 or IL-15 to Ly49H^+^ NK cells to promote their specific expansion and how XCR1^+^ DC contribute to this function has not been rigorously dissected.

## Role of DC subsets in the regulation of antiviral CD8 T cell responses to MCMV infection

**Figure 3:**
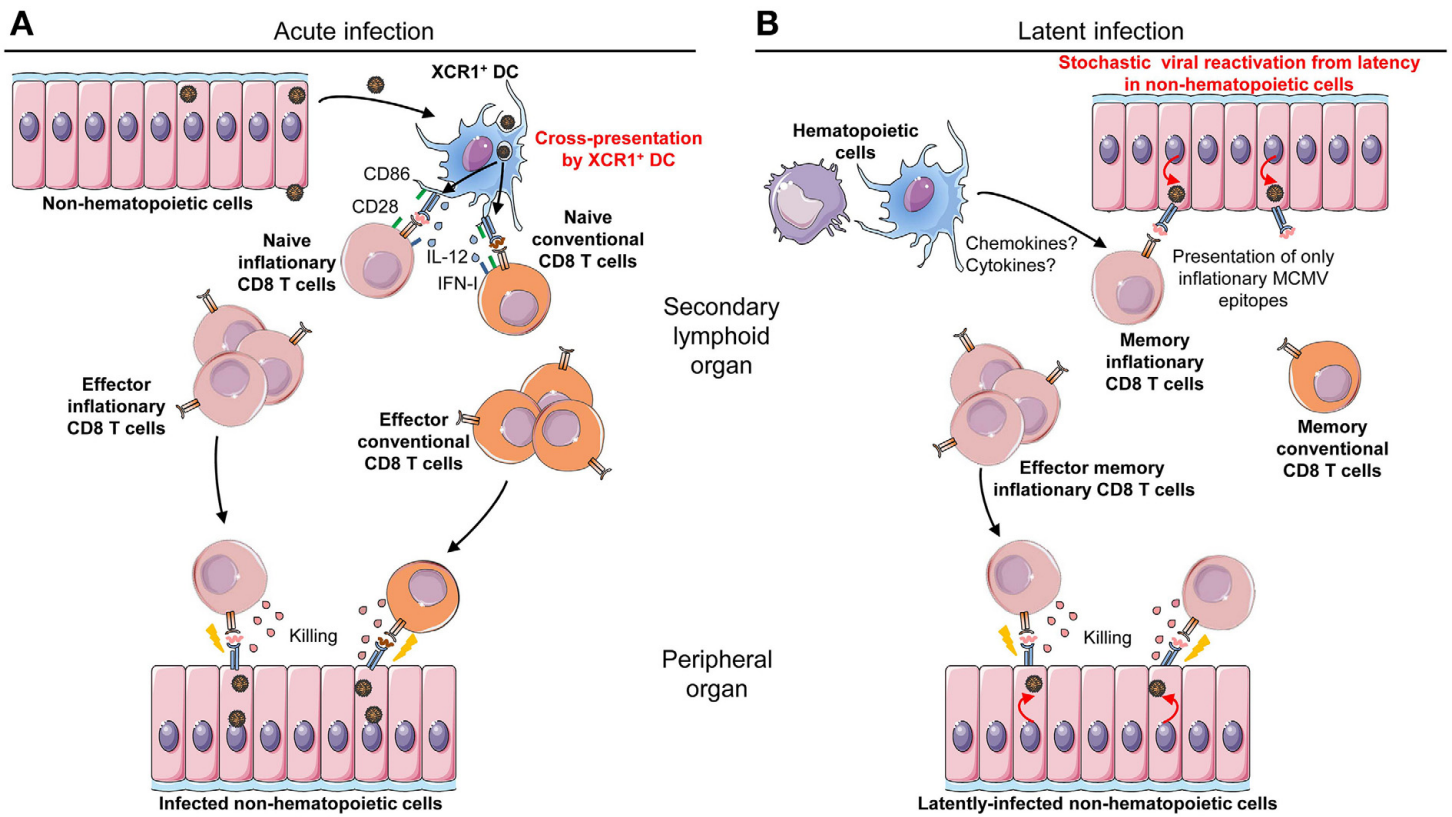
**Requirements for different antigen-presenting cell types during acute vs. latent MCMV infection. (A)** Professional cross-presenting XCR1^+^ cDC are necessary and sufficient for priming of MCMV-specific CD8 T cell during acute infection. XCR1^+^ cDC are able to take up and process antigenic proteins derived from infected cells, either non-hematopoietic cells as illustrated or the small fraction of infected DC. Once they have processed viral proteins into epitopic peptides, XCR1^+^ cDC can present them in association with MHC-I molecules (signal 1) to anti-MCMV CD8 T cells. The priming of naïve CD8 T cells for the induction of protective antiviral responses also requires activating co-stimulation signals (signal 2), such as the engagement of CD28 on the lymphocytes by CD80/CD86 expressed by the DC, and activating cytokines (signal 3) such as IL-12 or IFN-I. XCR1^+^ cDC are also competent for delivery of signals 2 and 3. Upon priming, naïve conventional and inflationary CD8 T cells differentiate into effector conventional and inflationary CD8 T cells, respectively, which control acute viral replication through recognition and killing of infected cells throughout the body. **(B)** Non-hematopoietic cells are necessary to drive inflationary anti-MCMV CD8 T cell responses during latent infection. After resolution of acute infection, the compartment of antiviral CD8 T cells contracts and gives rise to a low number of memory cells. In latent infection, MCMV can stochastically and transiently reactivate from latently-infected non-hematopoietic cells, causing the expression and presentation of a small number of viral antigens. This drives in turn the reactivation and proliferation of the memory CD8 T cell pool specific for the corresponding viral antigens. These CD8 T cells acquire an effector/effector-memory phenotype and expand continuously over time; a process called “memory inflation.” Even though hematopoietic cells are neither necessary nor sufficient for viral antigen presentation during latent infection, they might contribute to promote memory inflation by delivering other signals to CD8 T cells, such as cytokines or chemokines.

CD8 T cells significantly contribute to protection against MCMV infection (Reddehase et al., 1987; Lathbury et al., 1996; Polic et al., 1998; French et al., 2004, 2005; Mitrovic et al., 2012a). During acute MCMV infection, all antiviral CD8 T cells rapidly expand between days 4 and 8 after challenge. After, they undergo a contraction phase where they give rise to a low number of central memory cells which home to secondary lymphoid organs. After resolution of acute MCMV infection, once latency has been established, two different behaviors of antiviral CD8 T cells have been reported. Conventional central memory CD8 T cells self-maintain at constant levels. Inflationary CD8 T cells continuously expand over time with a significant fraction harboring an effector memory phenotype or an effector phenotype (Munks et al., 2006; Torti et al., 2011b). Inflationary CD8 T cells locate in non-lymphoid tissues such as the lung or the liver (Karrer et al., 2003; Torti et al., 2011b). This section will discuss the role of DC subsets in the priming, maintenance and reactivation of conventional and inflationary anti-MCMV CD8 T cells (Figure 3).

### Antigen presentation by non-hematopoietic cells or infected DC is dispensable to prime naïve CD8 T cells during primary MCMV infection

Infection of mice with viruses deleted of the MHC-I immune evasion genes m04, m06, and m152 does not affect the magnitude, repertoire and early kinetics of anti-MCMV CD8 T cell responses in C57BL/6 mice (Gold et al., 2004; Munks et al., 2007). Hence, rescuing antigen processing and presentation in infected cells *in vivo* does not increase the anti-MCMV CD8 T cell responses which are already remarkably high in mice infected with WT viruses. Hence, it was deduced that anti-MCMV CD8 T cells are not primed by infected cells but rather by cross-presenting cells which are not affected by viral immune evasion genes. In consistency with this hypothesis, XCR1^+^ DC isolated from the spleens of MCMV-infected C57BL/6 mice are not productively infected by MCMV but efficiently prime naïve anti-MCMV CD8 T cells *ex vivo* without the need to add exogenous antigen (Busche et al., 2013). In contrast, CD11b^+^ DC isolated from the same mice are productively infected and fail to activate naïve anti-MCMV CD8 T cells in *ex vivo* culture, unless they are pulsed with exogenous optimal epitopic peptides. Hence, productive MCMV infection of CD11b^+^ DC *in vivo* compromises their ability to directly process and present endogenous antigens to CD8 T cells, including viral proteins. The priming of anti-MCMV CD8 T cells must depend on XCR1^+^ DC cross-presentation of exogenous viral antigens that they have acquired from neighboring infected cell (Figure 3A).

### Professional cross-presenting XCR1^+^ DC are necessary for efficient priming of naïve anti-viral CD8 T cells during primary MCMV infection

By using a spread-defective MCMV mutant which only undergoes one replication cycle and cannot egress from the first infected cells, Snyder et al. demonstrated that MCMV-specific CD8 T cells are efficiently primed *in vivo* even when infected cells are deficient for MHC-I (Snyder et al., 2010). Hence, cross-presentation is sufficient to induce normal priming of anti-MCMV CD8 T cells *in vivo*. Other approaches demonstrate that cross-presentation is actually necessary for efficient induction of anti-MCMV CD8 T cell responses *in vivo*. Mutant MCMV were engineered to express both the human papillomavirus E7 and the influenza virus NP CD8 T cell epitopes, by knock-in into two different regions of the reporter fluorescent protein GFP: the E7 epitope in the signal peptide which prevents cross-presentation and the NP epitope within the mature GFP which allows cross-presentation, or reciprocally. Upon infection of mice with these MCMV mutants, antiviral CD8 T cells were efficiently generated only against the epitope that could be well cross-presented, and not against the epitope that was only presented directly, irrespective of the identity of these epitopes (E7 or NP). Even with viruses defective for MHC-I immune evasion genes, no efficient CD8 T cell priming was observed against antigens that could only be presented directly (Busche et al., 2013). Finally, the priming of MCMV-specific CD8 T cells is strongly impaired in Batf3-deficient mice, which have a dramatic and specific decrease in the numbers of professional cross-presenting XCR1^+^ DC in all tissues (Torti et al., 2011b). Altogether, these studies show that cross-presentation is necessary and sufficient for the induction of antiviral CD8 T cell responses during MCMV infection *in vivo*, and that XCR1^+^ DC are responsible for this function (Figure 3A). Cross-presentation of viral antigens by uninfected XCR1^+^ DC is likely necessary for the host to counter the evasion strategies evolved by the virus to dampen in infected cells the function of antigen presentation, positive co-stimulation and activating cytokine delivery to CD8 T cells. However, antigen cross-presentation might not be required in all organs for local priming of CD8 T cells. Indeed, in the absence of competent DC, infected liver sinusoidal endothelial cells can directly prime antiviral CD8 T cells *in vitro* and *in vivo* (Kern et al., 2010). Infected liver sinusoidal endothelial cells may escape the effects of viral MHC-I and co-stimulation immune evasion genes because they express immediate-early MCMV genes but poorly support productive virus replication (Dag et al., 2013).

### During latent MCMV infection, inflationary memory CD8 T cells are maintained by latently infected non-hematopoietic cells

In contrast to the requirement for the priming of naïve anti-viral CD8 T cells during acute infection, latently infected-non-hematopoietic cells appear to be the key antigen-presenting cells driving memory CD8 T cell inflation during latent infection (Figure 3B). Indeed, professional cross-presenting XCR1^+^ DC are not necessary for the inflation of the majority of antiviral memory CD8 T cells during latency (Torti et al., 2011b). Non-hematopoietic cells, particularly endothelial cells, are a major site of MCMV latency. They promote inflationary CD8 T cell expansion, which is driven by stochastic events of viral reactivation (Figure 3B) (Simon et al., 2006; Seckert et al., 2009; Arens et al., 2011). In C57BL/6 mice, long-term expansion and maintenance of H-2K^b^-restricted M38-specific CD8 T cells requires H-2K^b^ expression by non-hematopoietic cells. Consistently, MCMV genomes are not detected in hematopoietic cells during latent infection (Torti et al., 2011a). Similarly, during latent infection in BALB/c mice, when H-2L^d^ is expressed only in hematopoietic cells, H-2L^d^-restricted IE1-specific CD8 T cells fail to undergo inflation, in contrast to H-2D^d^-restricted m164-specific CD8 T cells (Seckert et al., 2011). Why the requirements for specific types of antigen-presenting cells are different during priming and inflation of MCMV-specific CD8 T cells is still an open question. One explanation could be that the activation of naïve T cells requires stronger or more complex signals than the reactivation of memory or effector T cells. Hence, professional cross-presenting XCR1^+^ DC might be needed for priming because they are the only APC expressing a high enough density of viral epitope/MHC-I complexes and of activating co-stimulation molecules, together with a proper cocktail of activating cytokines (Figure 3A), since they are not subjected to the inhibitory functions of viral immune evasion genes. Indeed, the response of naïve and memory conventional anti-MCMV CD8 T cells, but not the expansion of inflationary CD8 T cells during early and latent infection, requires a functional B7/CD28 activating co-stimulation axis (Arens et al., 2011) In contrast, the CD70/CD27 co-stimulation pathway promotes enhanced activation of MCMV-specific CD8 T cells for both conventional and inflationary responses, during both acute and persistent infection (Welten et al., 2013). An alternative explanation could be that professional cross-presenting XCR1^+^ DC do not access viral antigens during latent infection, because of stochastic, very low and extremely transient reactivation of MCMV in immunocompetent individuals. In any case, it remains possible that hematopoietic cells could be involved in the maintenance and expansion of inflationary CD8 T cells during latent infection through other functions than antigen presentation (Figure 3B). In particular, they might produce cytokines or chemokines promoting the survival and the proliferation of inflationary CD8 T cells or their local recruitment at anatomical sites of virus reactivation. Finally, it should be noted that at least one type of anti-MCMV inflationary CD8 T cell responses, the one directed against the IE3 viral protein, seems to be dependent on cross-presentation since it is impaired in Batf3-deficient mice (Torti et al., 2011b). In summary, the following model can be proposed to explain the inflation of antiviral CD8 T cell responses during latent infection (Figure 3B). Non-hematopoietic cells that are infected during acute infection become the main site of latent MCMV infection. Upon infrequent, stochastic and very transient episodes of viral gene desilencing, they express a very restricted repertoire of viral antigens. This leads to the expansion and maintenance of only those CD8 T cells specific for the antigens encoded by the viral transcripts expressed in latency (Seckert et al., 2012). Why are only a few viral epitopes driving inflationary CD8 T cells responses? The repertoire of viral antigens expressed in latently infected cells appears to be mostly limited to immediate early genes possibly because these are the first desilenced upon viral reactivation. It is likely that the rapid recognition of these antigens by effector inflationary CD8 T cells immediately shuts down viral reactivation, preventing expression of other viral genes during latent infection in immunocompetent hosts. In favor of this hypothesis, genetic manipulation of MCMV IE1 protein to disrupt its immunodominant inflationary epitope without affecting its function leads to an increased detection of IE1 desilencing in the lungs of latently infected mice, to the induction of events of IE3 transactivator splicing and to a significant increase in the frequency of anti-m164 inflationary CD8 T cells (Simon et al., 2006). Another hypothesis to explain the different antigenic repertoire of conventional vs. inflationary anti-MCMV CD8 T cells is a differential usage of proteasome subunits between inflationary and other viral epitopes. Indeed, mice deficient for one of the three catalytic subunits of the immunoproteasome (LMP7) have a reduced number of anti-MCMV CD8 T cells but with a stronger alteration of the frequency of conventional as opposed to inflationary antiviral CD8 T cells (Hutchinson et al., 2011). The immunoproteasome is constitutively expressed by professional APC, whereas non-immune cells only express it under the instruction of IFN-γ or IFN-I. Hence, it is possible that during acute MCMV infection, cross-presenting XCR1^+^ DC which contain immunoproteasomes and constitutive proteasomes stimulate responses to both conventional and inflationary epitopes. In contrast, in latent infection, only the epitopes produced by the constitutive proteasome would be presented by non-hematopoietic cells.

## Cross-talk between DC and NK cells modulate CD8 T cell responses during MCMV infection

**Figure 4:**
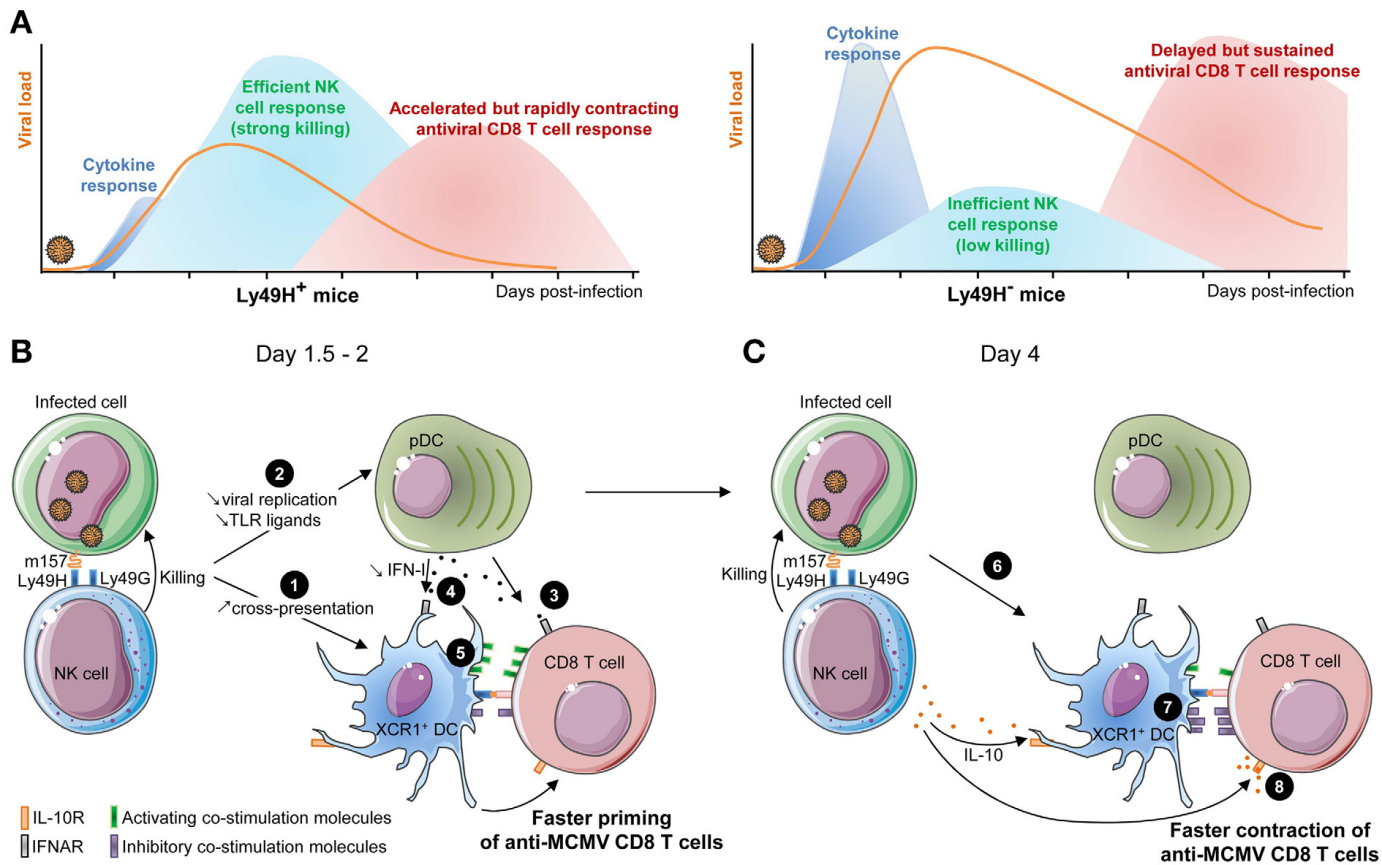
**Schematic model of the cross-talk between XCR1^+^ DC, NK cells, and CD8 T cells during MCMV infection. (A)** Comparative kinetics and intensities of innate cytokine, NK and CD8 T cell responses between mice having or lacking an efficient antiviral NK cell response. **(B)** Potential mechanisms in place early after infection of mice with efficient NK cell responses and promoting accelerated activation of anti-viral CD8 T cells. Early killing of infected cells may provide XCR1^+^ DC with a faster and increased access to viral antigen for cross-presentation by delivering them immunogenic apoptotic bodies (❶). It may also reduce the amount of viral ligands accessible to pDC and thus decrease systemic IFN-I production to promote its immune activation effects over the immunosuppressive ones (❷). Low IFN-I concentrations may limit the direct negative effects of these cytokines on CD8 T cells (❸), preserve the maintenance of high numbers of XCR1^+^ DC (❹), and promote an optimal maturation of XCR1^+^ DC by enabling them to selectively express high levels of activating but not inhibitory co-stimulatory molecules (❺). **(C)** Potential mechanisms in place later after infection of mice with efficient NK cell responses and promoting accelerated and stronger contraction of anti-viral CD8 T cells. NK cell killing of infected cells could rapidly shortage the supply of viral antigens available for cross-presentation by XCR1^+^ DC (❻). Cognate engagement of Ly49H by m157 leads to late IL-10 production by the NK cells which limit XCR1^+^ DC maturation (❼) and directly inhibit CD8 T cell proliferation (❽).

We will now discuss how the cross-talk between DC and NK cells impacts antiviral CD8 T cell responses (Figure 4).

### Efficient NK cell responses accelerate the induction of antiviral CD8 T cell responses

Mice able to rapidly control MCMV replication though Ly49H-dependent recognition and killing of infected cells by NK cells harbor an accelerated priming of naïve CD8 T cells. However, this response contracts earlier and never reaches the high levels observed in mice with an inefficient NK cell response (Figure 4A). Specifically, mice with an efficient NK cell response harbor a significant number of effector cytotoxic CD8 T lymphocytes in the spleen as early as 4 days post-infection, 24–48 h earlier than in Ly49H^−^ congenic animals (Figure 4A) (Robbins et al., 2007). Different mechanisms could contribute to this process (Figure 4B). Between days 1.5 and 2 after MCMV infection, in Ly49H^+^ mice, NK cells kill infected cells and may provide to XCR1^+^ DC faster and increased access to viral antigen for cross-presentation by delivering them immunogenic apoptotic bodies (Figure 4B, ❶). NK cells might also promote IL-12 production by XCR1^+^ DC, likewise to the mechanism proposed for improved induction of anti-tumoral CD8 T cell response in mice transplanted with NK cell-sensitive as opposed to NK-cell resistant tumors (Diefenbach et al., 2001; Adam et al., 2005). As compared to Ly49H^−^ mice, Ly49H^+^ animals show a strong decrease in serum levels of innate inflammatory cytokines, such as pDC-derived type I IFN and IL-12 (Figure 4A), and a better preservation of the compartment of professional cross-presenting XCR1^+^ DC. The induction of high systemic levels of IFN-I at the time of antiviral CD8 T cell priming by DC might be counterproductive for the host by negatively impacting the induction of adaptive cellular immunity. Hence, the early killing of infected cells by NK cells may reduce the amount of viral ligands accessible to pDC and thus decrease systemic IFN-I production (Figure 4B, ❷) to promote the immune activation effects of the cytokines over their immunosuppressive ones. Yet, it is not clear whether IFN-I dampen CD8 T cell priming through cell-intrinsic effects (Figure 4B, ❸) or indirectly by affecting XCR1^+^ cDC (Figure 4B, ❹). Both mechanisms might be operating. Indeed, exogenous injection of IFN-I in Ly49H^+^ mice recapitulates the striking delay in anti-MCMV CD8 T cell priming observed in Ly49H^−^ animals, but only causes a 2-fold decrease in the numbers of XCR1^+^ cDC, suggesting some direct inhibitory effects of IFN-I on CD8 T cells, as reported in other experimental models (Bahl et al., 2006; Marshall et al., 2011). However, the decrease in XCR1^+^ cDC numbers caused by IFN-I administration also probably affects the priming of anti-MCMV CD8 T cells. Low levels of IFN-I may be sufficient to induce an optimal maturation of XCR1^+^ DC by promoting their induction of the activating co-stimulation molecules CD40, CD80 and CD86 but not of the inhibitory co-stimulation molecules PD-L1 and PD-L2 (Figure 4B, ❺) (Vu Manh et al., 2013). Other experimental models were designed to compare anti-MCMV CD8 T cell responses of mice on the same genetic background but differing in the ability of their NK cells to control viral replication. They independently confirmed that efficient NK cell responses accelerate the induction of anti-MCMV CD8 T cell responses (Slavuljica et al., 2010; Stadnisky et al., 2011), although differences were noted depending on the genetic background of the mice in one study (Slavuljica et al., 2010).

### Efficient NK cell responses lead to an earlier contraction of antiviral CD8 T cell responses

Although an efficient NK cell response accelerates the priming of anti-MCMV CD8 T cells, several reports have shown that it also leads to an earlier contraction of this cellular adaptive immune response as early as 6 days post-infection (Figure 4A) (Robbins et al., 2007; Andrews et al., 2010; Mitrovic et al., 2012a,b). The sustained and stronger activation of anti-MCMV CD8 T cells observed in mice lacking efficient antiviral NK cells likely results from their failure to rapidly control viral replication such that *in vivo* expression of viral antigens is prolonged resulting in sustained priming of antiviral CD8 T cells (Andrews et al., 2010; Mitrovic et al., 2012a). Indeed, starting 4 days after infection, the total DC isolated from Ly49H^−^ mice prime more naïve CD8 T cells *in vitro* than the DC isolated from Ly49H^+^ animals DC (Andrews et al., 2010). In addition to an earlier shortage in viral antigen for cross-presentation by XCR1^+^ DC (Figure 4C, ❻), other mechanisms could contribute to the faster and stronger contraction of anti-MCMV CD8 T cells in mice with efficient NK cell responses. This includes late IL-10 production by the NK cells that had engaged in cognate interactions with infected cells, since IL-10 could inhibit the maturation of XCR1^+^ DC (Figure 4C, ❼) and directly inhibits CD8 T cell proliferation (Figure 4C, ❽).

### The cross-talk between DC and NK cells may act as a rheostat modulating the activation of anti-viral CD8 T cell responses commensurate to the threat caused by the level of MCMV replication.

In mice lacking efficient NK cell responses, strong anti-MCMV CD8 T cell responses are necessary to allow control of viral replication and to prevent lethal virus-induced cytopathic damages to vital organs such as the liver (Lathbury et al., 1996; Lee et al., 2009; Mitrovic et al., 2012a). In Ly49H^+^ mice, adaptive immune responses are necessary to ensure control of virus replication through different, complementary mechanisms, to avoid selection of NK cell-escape MCMV mutants (French et al., 2004, 2005). However, much lower levels of CD8 T cell responses are likely sufficient for this function, while maintenance of strong effector anti-MCMV CD8 T cell responses might lead to some immunopathology. Indeed, exacerbated production of TNF and IFN-γ by anti-MCMV CD8 T cells in the liver of MCMV-infected mice can induce hepatitis and ultimately cause the death of the animals (Livingston-Rosanoff et al., 2012). This CD8 T cell-dependent immunopathology can be prevented by immunoregulatory functions of NK cells (IL-10 production) (Lee et al., 2009) and of activated classical monocytes/MoDC/MDSC (nitric oxide production) (Daley-Bauer et al., 2012). Hence, both by controlling the overall viral antigenic load *in vivo* and by tuning the functions of DC, NK cells may act as a rheostat modulating the activation of anti-viral CD8 T cell responses commensurate to the threat caused by the level of MCMV replication, to promote the level of adaptive immunity sufficient for efficient control of the virus while limiting immunopathology.

## Conclusion and perspectives

This review aimed at giving a global overview of the current knowledge about the interplay between DC and MCMV *in vivo*, and how it could be interpreted to learn what makes successful immune responses against intracellular pathogens. Like all herpes viruses, MCMV has evolved a plethora of immune evasion genes including some interfering with DC functions. However, MCMV infection is inducing one of the most long-lasting and protective cellular adaptive immune responses known for an intracellular pathogen. This apparent paradox raises the question of the role of DC in the induction of protective immunity against MCMV, as well as whether and how the viral immune evasion genes could benefit both the host and the virus. The corresponding key issues we discussed are summarized in Table 2.

**Table 2:** **Key issues**.

MCMV is DNA β-herpes virus establishing life-long persistent infections in mice. It recapitulates many physiopathological characteristics of human CMV infection.
Dendritic cells (DC) are mononuclear phagocytes involved in both innate and adaptive immunity. They can be divided into two different subsets: plasmacytoid DC (pDC) and conventional DC (cDC). cDC are constituted by CD11b^+^ cDC and XCR1^+^ cDC. XCR1^+^ cDC are especially efficient in the activation of CD8 T cells, in particular through cross-presentation.
MCMV infection induces very strong and protective antiviral cellular immune responses *in vivo*. MCMV induces functional paralysis of DC *in vitro* but not *in vivo*. Several factors can contribute to explain this apparent discrepancy. The nature of the DC studied *in vitro* differs from that of the DC responsible for the induction of anti-MCMV adaptive immune responses *in vivo*. *In vitro*, most DC are infected, due to the use of very high MOI. Infected DC are unable to efficiently activate antiviral T cells, because of cell-intrinsic effects of MCMV immune evasion genes. These genes prevent processing and presentation of viral antigens, expression of activating co-stimulation molecules, and production of activating cytokines. *In vivo*, only a very small fraction of DC is infected. Hence, MCMV immune evasion genes do not strongly modulate overall DC functions *in vivo*. *In vivo*, the maturation of DC and the acquisition of their protective antiviral functions are promoted by the inflammatory milieu, in particular IFN-I, and by cross-talk with other immune cells, including NK cells.
*In vivo*, pDC are the main producers of IFN-I and IL-12, in response to triggering of TLR9 and TLR7 by MCMV-derived nucleic acid sequences.
The production of IFN-I, IL-15, and IL-12 by DC promote NK cell antiviral activity during MCMV infection. For efficient innate control of MCMV infection, NK cell also require direct recognition of infected cells through the engagement of activating receptors and/or through missing-self sensing.
During acute infection, naïve anti-MCMV CD8 T cells are primed by uninfected professional cross-presenting XCR1^+^ DC. Cross-presentation of viral antigens by XCR1^+^ DC is necessary to counter viral immune evasion strategies. However, in the absence of competent DC, in some tissues, non-hematopoietic antigen-presenting cells are able to prime naïve CD8 T cells.
The cross-talk between DC, NK cells, and CD8 T cells may act as a rheostat modulating the activation of anti-viral CD8 T cell responses to promote health over disease. Notably it can prevent irreversible damages to vital tissues as could occur due to either virus cytopathic effects or unbridled host immune responses.
CMV infection has the extraordinary property to induce strong, long-lasting, protective inflationary effector memory CD8 T cell responses. The inflation of memory anti-MCMV CD8 T cell responses is driven by recurrent episodes of stochastic and transient viral reactivation in latently infected non-hematopoietic cells. Viral antigen presentation by DC is neither sufficient nor necessary for this response.
Both cross-presenting XCR1^+^ DC and virus immune evasion genes play a key role in setting a balance between CMV and their hosts that may benefit both parties in the absence of developmental, genetic or acquired immunodeficiency of the host.
Owing to its unique ability to induce inflationary memory CD8 T cell responses, CMV has been successfully used as a vector for vaccines against chronic infections by difficult-to-treat intracellular pathogens in non-human primate disease models.

Contrary to early reports, it is now quite clear that there is no induction of global DC functional paralysis by MCMV *in vivo*. Additionally, the small fraction of the DC that are infected by MCMV does not exert a strong dominant immunosuppressive functions *in vivo*. On the contrary, MCMV infection very potently activates DC *in vivo*, in part due to the induction of high levels of IFN-I which promote DC maturation and their protective functions against disease and death; and also potentially due to mutually activating interactions between DC and NK cells. It is likely that this remarkable activation of DC *in vivo* by MCMV bears a significant contribution to the balance that has been reached between this virus and its host during millions of years of co-evolution. This balance results in a tight control of both virus replication and host immune responses, which preserves the health of immunocompetent hosts while still allowing establishment of viral latency and horizontal virus transfer.

The interaction between MCMV and its immunocompetent natural host, the mouse, appears to be precisely tuned to allow viral persistence and vertical spread. This includes the establishment of latent infection thanks to viral immune evasion genes, but no significant morbidity thanks to the host ability to mount the best suited level of antiviral immunity. On the one hand, host immune responses are strong enough to control viral replication in a manner preventing disease development consecutive to viral cytopathic effects. On the other hand, host immune responses are tightly controlled to preventing disease development consecutive to immune-mediated damage to essential organs. Astonishingly, although pDC are the major producers of IFN-I early after MCMV infection, they appear to be largely redundant for efficient control of the infection in most organs except in the SG (Swiecki et al., 2010). SG may play a role in the establishment of viral latency and constitute the most important site of MCMV horizontal transfer between hosts. The contribution of pDC to the control of MCMV latency remains to be investigated but might be significant since IFN-I plays an important role in this function (Dag et al., 2014). The delicate balance reached between MCMV and its host appears to be built on a critical cross-talk between professional cross-presenting XCR1^+^ DC and NK cells. This cross-talk operates as a rheostat to tune the kinetics, intensity and duration of effector anti-MCMV CD8 T cell responses, commensurate to the threat caused by the level of viral replication. In this regard, an intriguing question still open is the biological significance of the infection of a small fraction of XCR1^+^ DC by MCMV. An interesting possibility to consider is that it could promote cross-presentation of MCMV antigens by uninfected XCR1^+^ DC, by allowing their close proximity to infected cells in adequate micro-anatomical compartments, since infected and non-infected XCR1^+^ DC should co-localize *in vivo*. The balance reached between MCMV and its host also results from stochastic and very transient episodes of partial viral reactivation during persistent latent infection. These reactivation episodes act as “spontaneous vaccinal boosts” and drive continuous inflation of protective antiviral effector memory CD8 T cell responses.

Based on many studies discussed in this review, it seems that the immune evasion functions of MCMV might paradoxically benefit immunocompetent hosts through different mechanisms: (i) enhancement of the priming of CD8 T cells, by facilitating cross-presenting DC physical access to viral antigens, and by limiting the negative feedback caused by the killing of infected cells (Bohm et al., 2008), (ii) prevention of an exacerbated activation of CD8 T cells that could lead to severe immunopathology, (iii) establishment of latent infection leading to the induction of continuously expanding and long-lasting effector memory CD8 T cells and to a higher state of vigilance of innate immune cells. These adaptive and innate immune cells might be protective against virus reactivation from latency (Simon et al., 2006) and also against secondary infections by some other pathogens (Barton et al., 2007). This delicate balance between the virus and the host can be strongly perturbed in individuals with an immature or compromised immune system, where the functions of the viral immune evasion genes are not properly counterbalanced by the host immune responses. Most of these characteristics are expected to be shared by HCMV infection of humans. It should not be forgotten that HCMV is the virus the most frequently transmitted to the developing fetus. Congenital or neonatal infections often lead to severe morbidity or even to death. In addition, HCMV is a common opportunistic agent causing severe health problems in immunocompromised individuals, such as recipients of bone marrow or solid organ transplants, or AIDS patients. In these pathological contexts, the immune evasion functions of HCMV must clearly be deleterious to the host. It is likely that the functions of XCR1^+^ cross-presenting DC and their cross-talk with NK cells are compromised in immunosuppressed patients, adding up to the deficiencies directly affecting CD8 T cells and humoral immunity, and contributing to the failure to control HCMV. In addition to chemotherapeutic inhibition of the virus and to direct manipulation of antiviral CD8 T cell responses, treatments should perhaps aim also at rapidly restoring the functions and numbers of XCR1^+^ DC and NK cells in immunocompromised patients. This might be achieved by *in vivo* administration of FLT3-L and by delivering adequately adjuvanted HCMV antigens to XCR1^+^ DC.

In conclusion, CMV infection has the extraordinary property to induce strong, long-lasting, protective inflationary effector memory CD8 T cell responses. One the one hand, this property results from the ability of the host to mount strong innate immune responses to the virus. This involves the activation of professional cross-presenting DC for priming of CD8 T cells during acute infection, in the face of viral immune MHC-I evasion strategies. On the other hand, this property also results from the ability of the virus to establish latent infection, despite the powerful immune responses of the host, such that multiple episodes of viral gene desilencing occur throughout the lifetime of the host, boosting his immune responses. These unique properties of the interaction between CMVs and their hosts have led to the idea of using CMV as a vector for vaccines against chronic infections by difficult-to-treat intracellular pathogens. Several preclinical studies have been carried out in non-human primates, by vaccinating monkeys with a rhesus macaque CMV vector coding for proteins of the Simian Immunodeficiency Virus (SIV) (Hansen et al., 2009, 2011, 2013). Such vaccines are able to protect under prophylactic settings an important proportion of macaques against primary infection, by inducing a high frequency of CD4 and CD8 effector memory T cells able to home in mucosa and to control SIV before irreversible systemic spread (Hansen et al., 2011). These vaccines also protect under therapeutic settings, leading to the elimination of SIV in macaques where systemic viral dissemination had already occurred. In infected macaques therapeutically vaccinated with a CMV vector expressing SIV antigens, both replicative virus and proviruses become undetectable in all tissues examined. This viral clearance takes months to be effective, suggesting that it occurred through continuous immune surveillance of signs of SIV reactivation by CMV vaccine-induced inflationary effector memory T cells. In case of reactivation, these effector memory CD8 T cells could eliminate immediately the latent cellular reservoirs of SIV (Hansen et al., 2013). The new challenge ahead would be to further advance our knowledge of the interaction between CMV and the immune system to design alternative synthetic vaccines able to induce such strong and protective responses without using viral vectors, if ever possible.

### Conflict of interest statement

The authors declare that the research was conducted in the absence of any commercial or financial relationships that could be construed as a potential conflict of interest.
